# Towards minimally-invasive, quantitative assessment of chronic kidney disease using optical spectroscopy

**DOI:** 10.1038/s41598-019-43684-8

**Published:** 2019-05-09

**Authors:** Mostafa E. Belghasem, Ousama A’amar, Daniel Roth, Joshua Walker, Nkiruka Arinze, Sean M. Richards, Jean M. Francis, David J. Salant, Vipul C. Chitalia, Irving J. Bigio

**Affiliations:** 1 Department of Pathology and Laboratory Medicine, Boston University School of Medicine, Boston, MA USA; 2 Department of Biomedical Engineering, Boston University, Boston, MA USA; 3 Renal Section, Department of Medicine, Boston University School of Medicine, Boston, MA USA; 4 Department of Surgery, Boston University School of Medicine, Boston, MA USA; 5 Department of Electrical & Computer Engineering, Boston University, Boston, MA USA; 6 Veterans Administration Boston Healthcare system, Boston, MA USA

**Keywords:** Sensors and probes, Chronic kidney disease, Biomedical engineering, Optical sensors

## Abstract

The universal pathologic features implicated in the progression of chronic kidney disease (CKD) are interstitial fibrosis and tubular atrophy (IFTA). Current methods of estimating IFTA are slow, labor-intensive and fraught with variability and sampling error, and are not quantitative. As such, there is pressing clinical need for a less-invasive and faster method that can quantitatively assess the degree of IFTA. We propose a minimally-invasive optical method to assess the macro-architecture of kidney tissue, as an objective, quantitative assessment of IFTA, as an indicator of the degree of kidney disease. The method of elastic-scattering spectroscopy (ESS) measures backscattered light over the spectral range 320–900 nm and is highly sensitive to micromorphological changes in tissues. Using two discrete mouse models of CKD, we observed spectral trends of increased scattering intensity in the near-UV to short-visible region (350–450 nm), relative to longer wavelengths, for fibrotic kidneys compared to normal kidney, with a quasi-linear correlation between the ESS changes and the histopathology-determined degree of IFTA. These results suggest the potential of ESS as an objective, quantitative and faster assessment of IFTA for the management of CKD patients and in the allocation of organs for kidney transplantation.

## Introduction

Close to 10 million Americans have chronic kidney disease (CKD)^1^, which is characterized by the presence of tubulointerstitial fibrosis (IF) and tubular atrophy (TA) (IFTA). IFTA is a universal pathologic feature implicated in the progression of CKD, irrespective of inciting cause^2,3^ and correlates strongly with renal survival in humans and also guides therapeutic decisions^4^. Thus, accurate quantification of IFTA is clinically important, yet current methods of estimating IFTA have several limitations. Ultrasonic measurement of kidney size and the echo texture is used for such purposes, but it is a crude tool that only becomes sensitive when the kidneys have already begun to shrink in size and become denser because of tubular atrophy and replacement with fibrous tissue. Elastography, a technique that has proven valuable for detecting and quantifying liver fibrosis, cannot be used to detect and measure kidney fibrosis because the kidneys, as opposed to the liver, are deeply embedded in the abdominal cavity, with thick muscle layers behind and bowel in front. While several MRI-based methods such as diffusion-weighted MRI, BOLD MRI, MR elastography, and renal susceptibility imaging have been investigated to assess severity of renal fibrosis, these techniques have limited accuracy or are confounded by the deep position of the kidneys in the retroperitoneal space covered by muscles, resulting in contradictory results^5^.

Among the options, an IFTA estimate derived from histological assessment of a kidney biopsy is widely accepted as the gold standard and is the current clinical practice. This method is slow, labor-intensive and fraught with variability and sampling error. It provides a semi-quantitative estimate, which is derived from one component of fibrosis, collagen-I, detected with the use of stains such as Masson Trichrome. In addition to variability in staining technique, the method commonly suffers from a lag time of ~48 hours due to tissue processing. This delay is undesirable, particularly in the setting of kidney transplantation, where the decision of accepting and implanting a kidney allograft from a deceased donor depends on a rapid and on-site assessment of fibrosis. Moreover, an IFTA estimate is derived from 1–3 core biopsies of a kidney and is assumed for the entire kidney. This extrapolation introduces sampling error, as IFTA can be patchy within the kidney. While a determination of IFTA at multiple regions in renal parenchyma would provide a better estimate of the extent of renal damage, it is not feasible with core biopsies because of the risk of bleeding induced by core biopsies^6^. Moreover, the semi-quantitative method currently used to measure fibrosis is subjective, quite imprecise and observer-dependent. Whereas interstitial fibrosis measurements derived from detailed scoring systems, including whole-slide digital images of kidney biopsies, have been shown to predict outcome^7–9^ this is time-, resource- and labor-intensive. Thus, there is an unmet need for a universal, minimally invasive, accurate, rapid, quantitative and standardized method for measuring IFTA for routine clinical prognosis and to guide therapy and stratify subjects for interventional studies.

Optical technologies offer the potential to meet that need. To date, however, none of the published reports using optical technologies have focused on the potential for clinical translation. One group has recently shown that molecular vibrational spectroscopy can detect spectral changes associated with peptide modes in collagen (mainly C–O and C–O–C stretch modes), as indicative of increasing amounts of collagen^10^, but this method requires biopsy sample preparation for off-line infrared spectroscopic microscopy (analogous to histopathology but without staining) and cannot be used in real time or for *in-vivo* applications. Other optical methods that have been reported include fluorescence lifetime imaging^11,12^ and second-harmonic microscopy^13,14^, but these suffer from the same limitations, are very expensive, and do not offer clinical translation.

The method reported here, elastic-scattering spectroscopy (ESS) offers the potential for objective, quantitative, rapid, clinical application at low cost, based on optical measurements of unlabeled tissue. Bigio and colleagues developed the original methods of ESS, and demonstrated its clinical applications for noninvasive tissue diagnosis, based on the scattering properties of cellular and subcellular structures, predominantly for detecting cancer and pre-cancer. (See, for example, early demonstrations^15–17^) ESS has shown promise as a minimally-invasive tool for distinguishing a variety of disease pathologies in organs such as urinary bladder^16^, breast and associated lymph nodes^18–20^, prostate^21^, thyroid^22,23^ and pathologies in the GI tract, such as dysplasia in the esophagus^24–28^, polypoid neoplasia in colorectal cancer screening/surveillance^29–32^ and colitis and dysplasia in patients with IBD^17,29,33^.

## Results

**Figure 1:**
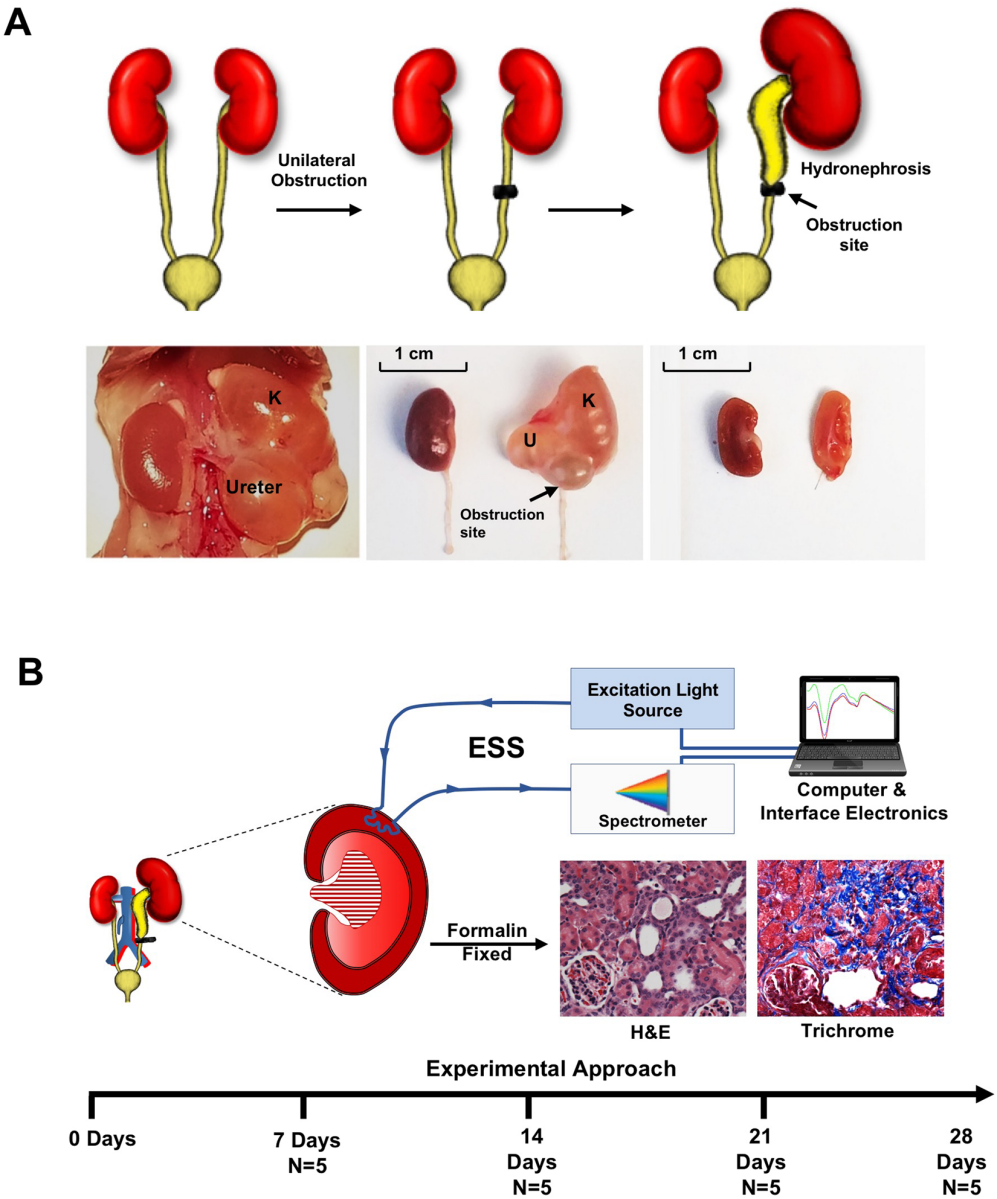
(**A**) Schema of generation of the UUO model. (Upper panel) C57BL/6 mice underwent laparotomy to ligate left ureter, and were allowed to progress for times up to 4 weeks. The obstructed kidneys develop hydronephrosis and hydroureter, while the contralateral kidney hyperfunctions to compensate for the partial loss of functional renal mass. Lower panel left: displays the normal right kidney and left hydroureter and hydronephrotic (K) kidneys. Lower panel middle: shows the relative sizes of the obstructed kidney compared and the normal contralateral kidney. Lower panel right: the kidneys were sliced open at the hilum, and their internal surfaces are shown. The obstructed kidney is pale colored with thinner parenchyma. (**B**) Schema of experimental strategy. Two different models of CKD were performed. Fresh kidneys after harvest were subjected to ESS and then processed by formalin fixation and then stained using H& E and Trichrome. Blood was analyzed for BUN to assess renal function.

From among several available animal models of CKD, we examined ESS spectra in two well-established rodent models^34,35^, which are characterized predominantly by interstitial fibrosis and tubular atrophy (IFTA). The unilateral ureteric obstruction (UUO) model, a model of obstructive nephropathy in humans, was generated by ligating the left ureter in a group of C57BL/6 male and female mice (Fig. 1A). As represented in Fig. 1B, groups of four mice were harvested at intervals of one week, for a total duration of four weeks. The renal function was measured by blood urea nitrogen (BUN), and the extent of IFTA was estimated on histology of the kidney tissue stained with Masson Trichrome stain.

**Figure 2:**
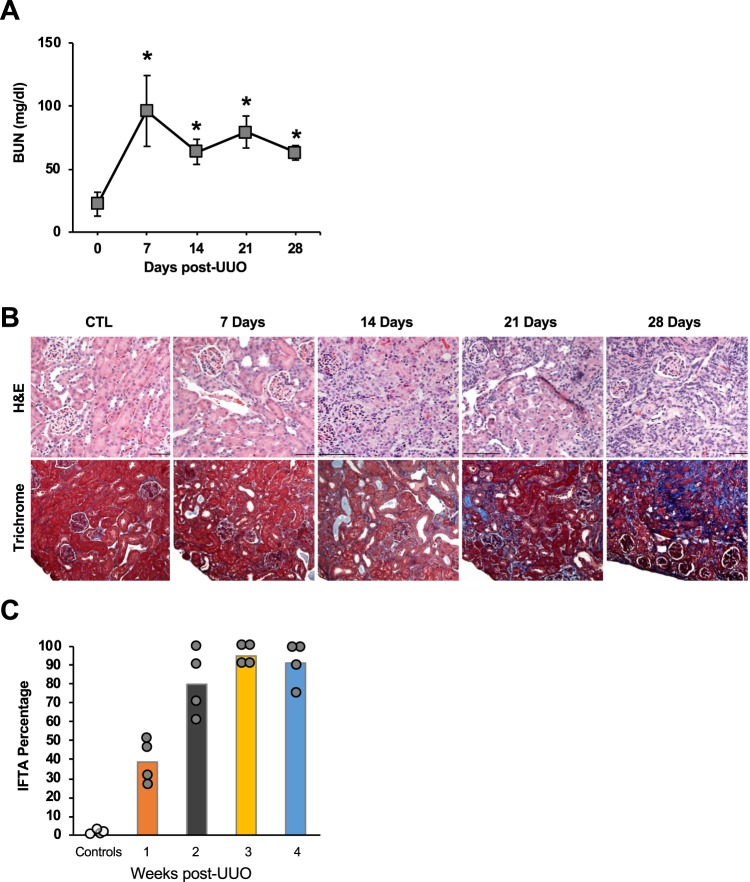
(**A**) Rise of BUN in animals with obstructed kidneys. Average BUN from four animals per time points is shown. Error bars = total range of values. (**B**) Kidney harvested from mice at every point were stained with Hematoxylin & Eosin (upper panel) and Trichrome stain (lower panel). Blue color of Trichrome stain corresponds to the fibrotic tissue. Gross specimens: scale bars = 1 cm. (**C**) IFTA percentage in UUO model. IFTA is represented as the percentage of tissue area showing fibrosis, as described in the Methods, as determined by a renal pathologist blinded to the sample. Average percentage per time-point (N = 4 animals/time-point) is shown, along with all values for individual animals.

The UUO model resulted in hydroureter and hydronephrosis on the left side with eventual thinning of the renal cortex (Fig. 1A). Blood urea nitrogen (BUN) levels were monitored weekly in mice to follow deterioration of renal function (Fig. 2A). While BUN levels were significantly higher in mice over a three-week period, compared to the control time, t = 0, the highest BUN was noted in the first week. The baseline average BUN (t = 0, average ± SEM) was 22.2 ± 4.2 mg/dl. BUN rose acutely and significantly in the first week post UUO to 96.1 ± 4.5 mg/dl; p < 0.05. This acute rise was followed by a reduction in the 2^nd^ week post UUO to 63.7 ± 4.5 mg/dl, and then BUN stabilized in the range of 79.4–62.9 mg/dl for last 2 weeks. This pattern of rise and stabilization of BUN after the first week of ureteric ligation represents acute tubular injury during the early phase of obstruction, followed by compensatory hyper-functioning of the contralateral right kidney, as described by others^36,37^. The average dimension of kidneys in the UUO model varies depending on the time elapsed after ureter obstruction. The obstructed kidney undergoes enlargement due to backpressure changes associated with the ureteric ligation, followed by damage to the cortex resulting in a thin cortex. At the end of 4 weeks, the obstructed kidneys for the UUO model had sizes (measured as length) of 12–13.5 mm.

The obstructed kidneys showed progressive increase in IFTA over the entire duration of obstruction. The kidneys harvested at end of 7 days showed prominent dilatation of tubules and loss of brush border, damage of both the proximal and distal tubular cells, and interstitial infiltrates. These acute changes were replaced by loss of glomeruli, tubular atrophy, and loss of tubular mass on H& E staining in kidneys from later time points (Fig. 2B). Masson Trichrome stain showed increasing amounts of fibrosis, seen as blue-colored stain, and highest in the kidneys harvested at the later time points. The blue color in the Masson Trichrome stained section indicate the deposition of collagen in the renal interstitium. The IFTA was semi-quantitatively evaluated by a nephropathologist using standard assessment of Trichrome stain density and distribution, on a scale of 1–5, with the value of 5 corresponding to IFTA of 100% of the parenchyma. This IFTA percentage exhibited a progressive increase with the duration of obstruction (Fig. 2C).

**Figure 3:**
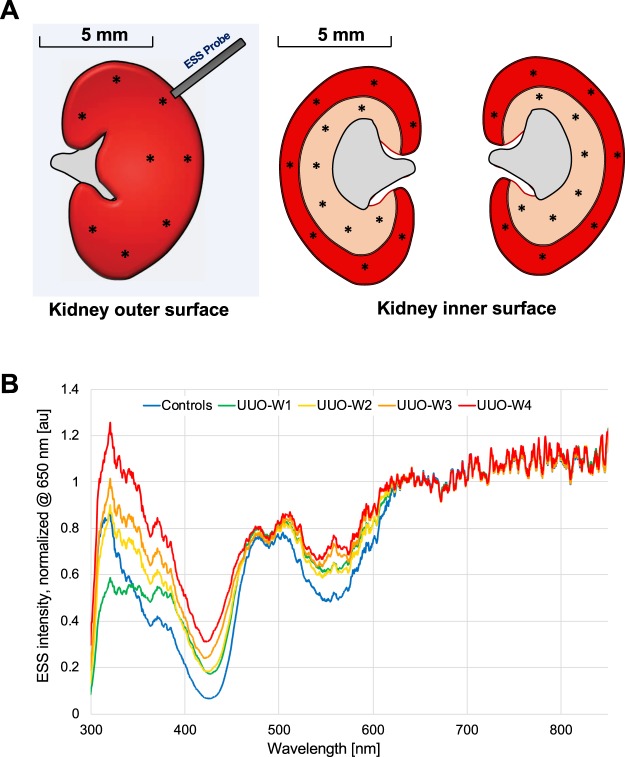
(**A**) Left: ESS spectra were obtained from a series of locations from outer surface of kidneys after removal of Gerota’s fascia. Right: The kidney was then sliced open (bisected), and ESS spectra were obtained from various locations of the cortex and medulla as shown. (**B**) Averaged spectra obtained from the inside (cortex and medulla) and surface of obstructed left kidney over four weeks duration are shown. Four animals per time point after induction of UUO model are compared with kidneys from control animals. The spectra are normalized at 650 nm to facilitate comparison of spectral shapes and relative spectral amplitudes at the shorter wavelengths.

Using a fiberoptic probe, the ESS spectra for the UUO model were recorded from several points on the external surface and from the inside of the bisected kidneys (Fig. 3A**)**. The ESS method and the fiberoptic probe design are described in more detail in the Methods section. Briefly, a small fiberoptic handheld probe integrates two adjacent optical fibers, one for illumination (with a short pulse of broadband light) and the other for collecting some of the light backscattered from the tissue. When recording an ESS spectrum, the tip of the probe is placed in gentle contact with the tissue surface, and the backscattered light is collected and recorded with a spectrometer.

The spectra, as plotted, were normalized to the value of 1 at 650 nm, to enable comparison of spectral shapes, independent of overall amplitude (Fig. 3B). The familiar Soret- and Q-bands of hemoglobin optical absorption are seen (at ~420 and 540–580 nm); and the depth of these bands correlates with blood-volume fraction. However, given that these measurements were made *ex-vivo*, without active perfusion, we ignored this feature and sought to address changes in optical diffuse reflectance as an indication of variations in the tissue scattering coefficient associated with relative amounts of collagen. Given the small dimensions of collagen fibrils^38^, increases in collagen content can be expected to enhance diffuse reflectance at shorter wavelengths, relative to longer wavelengths^39^. The readily-observed increases in optical reflectance at shorter wavelengths (350–550 nm) relative to the normalization point (650 nm) were found to correlate monotonically with the degree of IFTA **(**Fig. 3B**)**. For the analysis presented here, we chose (by inspection) the integrated area of the spectral range 350–450 nm as the optical biomarker for comparison with IFTA percentages from histopathology, given the stronger effects of scattering by collagen at shorter wavelengths.

Next, we confirmed the ESS spectral biomarker in another model of CKD. The adenine-diet-induced (AD) mouse model for CKD is a model of crystal-induced CKD in humans. It is characterized by increases in BUN and creatinine, hyperphosphatemia and systemic uremic toxicity, including secondary hyperparathyroidism, bone disease, and vascular calcification within two to four weeks of exposure to a diet supplemented with adenine^40–43^. Adenine is metabolized to 2,8-dihydroxyadenine and precipitates into crystals in the microvilli and apical epithelial region of the proximal tubules, causing tubular damage and IFTA^41^. The adenine-diet model is characterized by profound fibrosis, and some reduction in the size of the kidney. Compared to the kidneys of mice on normal chow (length range ~9–10.5 mm), those on adenine chow had kidney lengths in the range 8–9 mm

**Figure 4:**
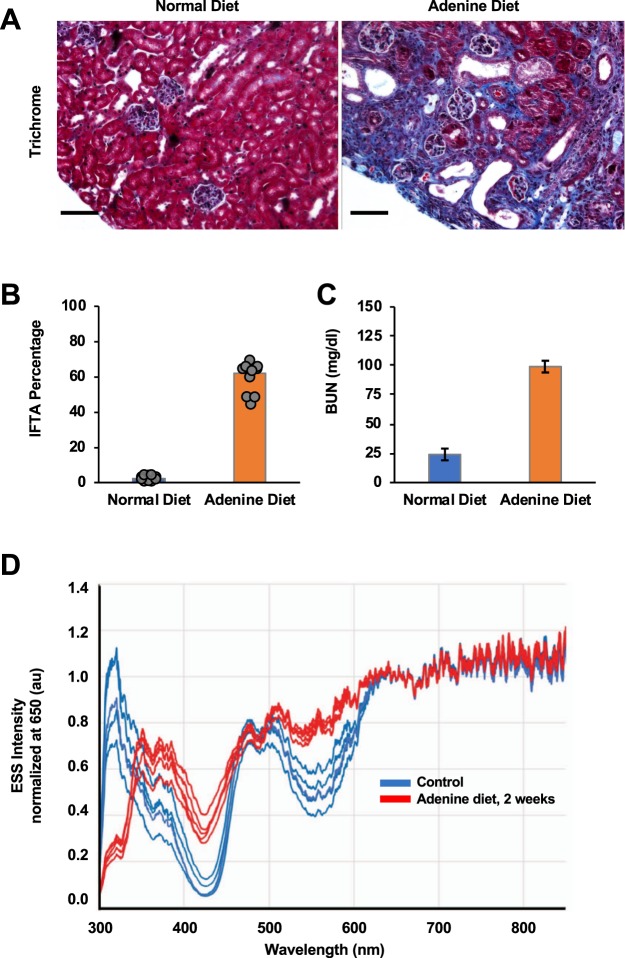
(**A**) Extensive fibrosis in adenine-fed mice. Kidneys of C57BL/6 mice exposed to 0.25% Adenine diet for one week followed by 0.2% diet for one week are shown and compared to those on normal chow. Histology images of kidney tissue stained with Masson Trichrome are shown. Scale bar = 100 µm. (**B**) IFTA percentage in the AD model. A percentage of IFTA (determined by histopathology with Masson Thrichrome staining) as described in Methods, determined by a renal pathologist blinded to the sample. Average percentages for controls and 10 treated mice (N = 5, for each of two strains) at 14 days are shown. Values for individual animals shown, with some overlap. (**C**) Significant rise in BUN in adenine-diet animals. Average BUN from five C57BL/6 controls and five AD mice at 14 days are shown. Error bars = full range of vales. (**D**) Individual spectra from 5 control mice and 5 adenine-diet C57BL/6 mice at 14 days. The spectra are normalized at 650 nm to facilitate comparison of spectral shapes. (Spectra from the 129/SvEV strain, not shown, were essentially similar). We note that, although spectral trends in the range 350–450 nm are essentially similar to those in the UUO model, the AD model exhibits an absorption band at wavelengths below 350 nm. We have not identified the source of this absorption, although we speculate that this may be due to the dihydroxyadenine crystals that form in the kidney, and that exhibit strong absorption in the UV-B region.

We examined this model in the C57BL/6 mice and subsequently confirmed our findings in the 129/SvEv mice. The results for both strains were substantially the same, and in Fig. 4 we show the data from C57BL/6 mice, as exemplary of the two. This strategy was employed to rule out strain-specific differences in the susceptibility to adenine-induced injury and ESS changes. In both strains, the experimental group consisted of a combination of male and female mice and received a diet containing 0.25% adenine for one week followed by 0.2% adenine for three weeks, while mice on normal chow served as controls. In the C57/BL6 mice, the adenine-fed group exhibited profound tubular atrophy, fibrosis, inflammatory cell infiltration, dilatation of tubules, intraluminal casts, all suggestive of loss of renal mass and IFTA (Fig. 4A). The IFTA percentages were significantly higher in the adenine-fed group (Fig. 4B). Extensive renal damage was corroborated by a significant rise in BUN in the animals with adenine diet at t = 14 days. (Normal chow: BUN 20.30 ± 1.92 mg/dl; t = 14 days, BUN = 104.6 ± 4.10 mg/dl, p < 0.001) (Fig. 4C). The 129/Svev mice exhibited similar trends to the C57/BL6 on an adenine diet (data not shown). The ESS spectra obtained on these animals (Fig. 4D) differ in shape from those observed in the fibrotic kidneys of the UUO model – the two methods do not generate precisely the same pathology – but they also exhibit increased signal in the range 350–450 nm compared to the control. For the AD model, Fig. 4D displays ESS spectra from individual animals in the C57BL/6 group, to illustrate the degree of variance.

**Figure 5:**
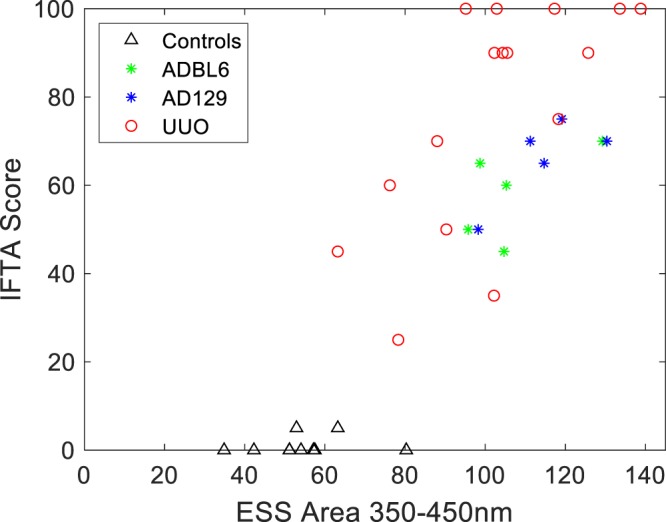
Comparison of the degree of IFTA from histopathology of all mice, presented as individual points, against a simplified ESS spectral feature chosen by inspection (the relative areas under the curve of the spectra from 350 to 450 nm). IFTA assessment of the stained kidney slides was performed by an expert nephropathologist, and is expressed as a percentage of total kidney area that was affected. Data are displayed for all time-points of the UUO model, together with the data of the AD model (at 14 days). Controls (untreated) for both models are represented by the triangles (and evidenced no significant difference between the two strains used in the AD model). Computed correlation coefficient: r = 0.85; confidence interval [0.73. 0.92]; p-value, p ≪ 0.05.

Figure 5 shows a scatter plot of the entire set of 40 animals, for both models (and both strains in the AD model), and the controls, comparing the ESS spectral biomarker (area under the curve from 350 to 450 nm) with the individual IFTA percentages derived from Masson Trichrome stain analysis by the nephropathologist. The computed correlation coefficient was r = 0.85, with a confidence interval [0.73. 0.92], and p-value, p ≪ 0.05.

## Discussion

While these results constitute an encouraging proof-of-concept, our motivation for the application of ESS is two-fold. First, in the short-term, ESS can provide rapid, objective (quantitative) assessment of fibrosis on biopsied kidney tissue. This *ex-vivo* assessment will fit well within the current clinical work flow of the patient and has high potential for rapid adoption. Second, this ESS method offers the future potential to be leveraged for *in-vivo* determination of renal fibrosis by incorporating the probe into a fine-needle tool (25-gauge), which would enable real-time transdermal clinical assessment of CKD, but with lower trauma than for the typical large-core biopsy tool.

The current work examines changes in ESS spectra in fibrotic kidneys of animals in two discrete CKD rodent models; its broad applicability requires validation in other animal models of CKD representing different types of human CKD, and also in human kidneys with different degrees of fibrosis. Our future work will include careful spatial registration of measurement locations on kidney cores, to have pathology readings that are co-registered reliably with the corresponding ESS spectral patterns, enabling development of more specific quantitative spectral biomarkers that correlate with staging of CKD. It is understood that perfusion will be different for *ex-vivo* vs *in-vivo* measurements. This study was intended to provide motivation for potential future studies with *in-vivo* protocols. As such, we compared our results to histopathology, which is also conducted *ex-vivo*. The resulting data indicate dramatic spectral changes due to changes in the scattering properties of fibrotic renal tissue, independent of differences in Hb absorption.

The potential advantages over the current practice are substantial. The standard-of-care practice uses a large (~16-gauge) core-biopsy tool, with an OD of 1.65 mm, and it extracts a tissue sample that is ~1 mm in diameter and ~15 mm long, limited to the superficial cortex. Due to the tissue trauma caused by such large biopsy tools, there is a substantial risk of bleeding; post-biopsy ultrasound frequently documents hematoma adjacent to the kidney^6^. Therefore, such cores are employed sparingly (typically 2–3 cores), which limits the areas and depth of kidney that can be sampled, a major limitation. In the future, once proven, attempts can be made to perform ESS measurements within the renal parenchyma using a 25-gauge ESS probe (0.51 mm diameter). While this method only partially circumvents the problem related sampling, such a technique may obviate the need for an excised tissue sample for diagnosis of IFTA, and can be expected to exhibit a dramatic reduction in tissue trauma and bleeding^44^, enabling more extensive sampling along a larger number of tracks, over the full length of each track, traversing from superficial cortex to deep medulla. The ESS spectrum is also sensitive to the strong absorption by oxy-/deoxy-hemoglobin, which provides additional diagnostic information, as the status of hemoglobin is dependent on renal perfusion: capillary rarefaction observed with renal fibrosis compromises the perfusion within renal parenchyma^45–48^. Thus, in addition to the spectral biomarkers for quantitative assessment of fibrosis, the changes in oxy-/deoxy-hemoglobin are likely to alter the ESS spectra acquired interstitially, and this can be used as an informative parameter to estimate renal parenchymal perfusion^49,50^.

Finally, we wish to note that fibrosis also figures prominently as a threatening factor associated with a number of other tissue pathologies, prominent among them being idiopathic pulmonary fibrosis (IPF)^51^. Traditional imaging methods are often unable to distinguish IPF from other lung abnormalities^52^, leading to the requirement for invasive surgical biopsy. Variations of the method reported here could enable low-cost, noninvasive and quantitative diagnosis of IPF, by optical measurements mediated by the new generation of smaller bronchoscopes that reach into more distal pulmonary branches^53,54^.

In summary, IFTA is the critical component of renal biopsy evaluation that has profound clinical implications for a patient from prognostic perspective and for therapeutic decision. The current gold-standard for IFTA evaluation has several limitations especially in certain clinical scenarios, such as donor kidney evaluation for the cadaver transplantation. Hence, developing rapid orthogonal techniques with high accuracy is imperative. While the current body of work illustrates a simplified differentiating biomarker extracted from the ESS spectra, it provides impetus for continued elaboration of the methods, for which we will develop rigorous algorithms based on the rich information content of the entire ESS spectral pattern, enabling development of quantitative morphometric indices of clinically relevant biomarkers for disease. In short, ESS has potential to address several limitations of the current standard-of-care. It indeed enables faster and more quantitative assessment of IFTA in extracted tissue, and, has the future potential for immediate bedside assessment of fibrosis and tubular atrophy.

## Methods

### Brief description of elastic-scattering spectroscopy (ESS)

Earlier applications of ESS addressed epithelial pathologies (pre-cancer and early cancer), wherein alterations in the cellular and sub-cellular architecture and ultrastructure are responsible for the changes in ESS spectra. Similarly, alterations in the extracellular matrix also affect the wavelength-dependence of elastically-scattered light, enabling ESS to be responsive to varying amounts of fibrosis. The main elements of the ESS method and instrumentation have been described in a number of earlier publications^17,55–58^.

**Figure 6:**
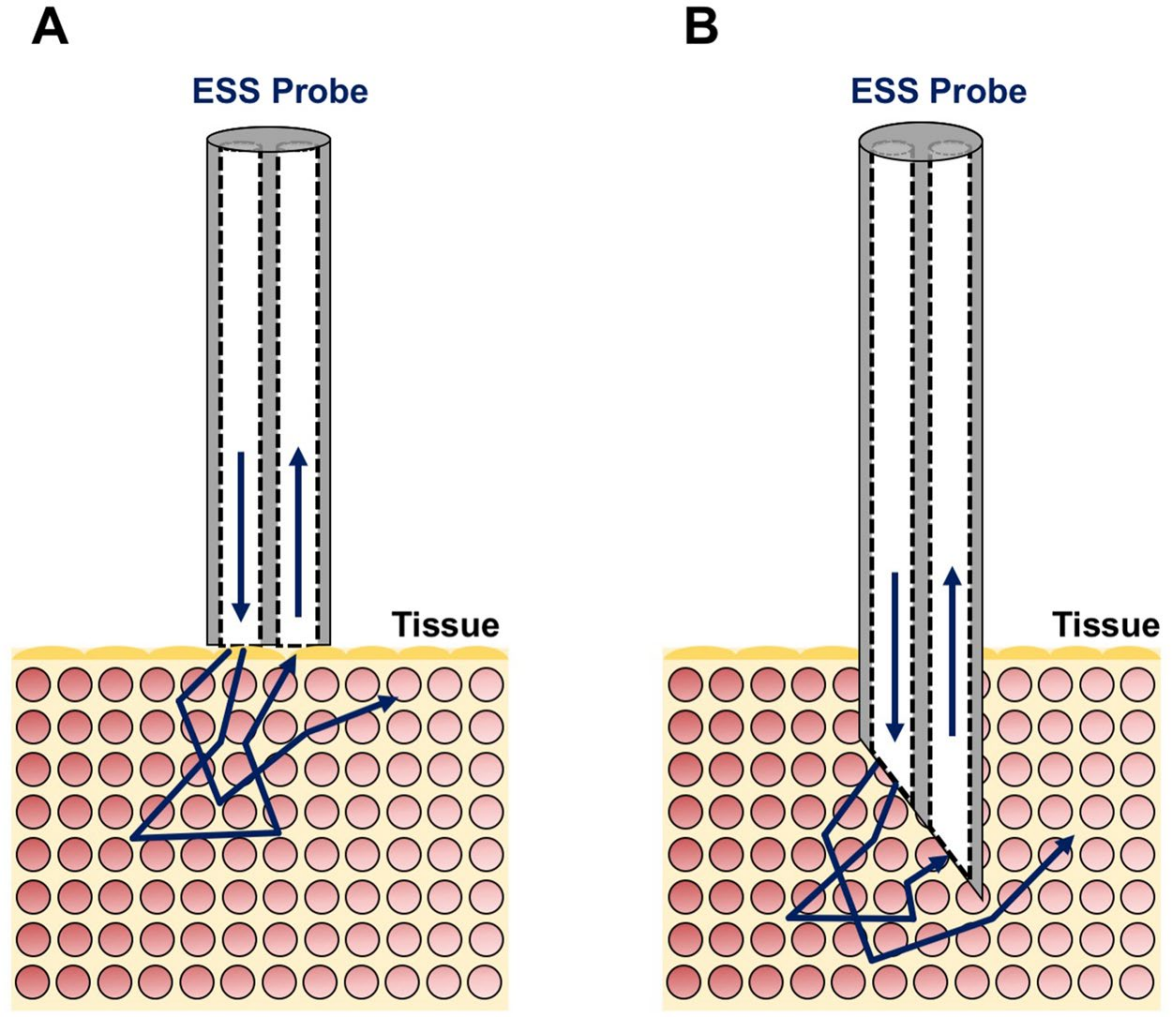
Diagrammatic representation of two options for the ESS fiberoptic probe geometry. (**A**) a square-ended probe for measurements at the surface of a tissue; (**B**) fiberoptic probe integrated into a fine needle for measurements inside solid organs. In this study, all of the data presented were generated with the geometry for surface interrogation. In future studies, especially for transdermal implementation, the needle geometry can be integrated with a long 25-gauge fine needle, to minimize tissue trauma.

In these implementations, ESS is an instant point-spectroscopic measurement technique, not an imaging modality, which measures the spectrum of backscattered light from tissue near the probe tip, over a broad wavelength range (320–900 nm in our current system), using a fiberoptic geometry that enhances sensitivity to the cellular and tissue architecture, and micromorphology of ECM structures. ESS is a form of sub-diffuse reflectance spectroscopy, specifically (and importantly) at very small source-detector separations (here, ~150 μm), for which the diffusion equation is not valid, and sensitivity to the scattering phase function is enhanced. In various clinical implementations, the tip of the probe (invoking separate illuminating and collecting fibers) is either in optical contact with the surface of the tissue under examination (Fig. 6A), or integrated into a fine needle and inserted into the parenchyma of solid organs (Fig. 6B). When performed with this optical geometry, ESS is sensitive to the absorption spectra of strong chromophores (e.g. oxy-/deoxy- hemoglobin) but, more importantly, measures scattering properties, such that the spectral features relate to sub-cellular structural features, as well as structures of the extracellular matrix.

The ESS control system is small (as small as a box of tissues), portable, and can be easily carried into the clinic. The light source is a pulsed (~20 μsec) xenon short-arc lamp (Hamamatsu, Inc.) and it is coupled to the illumination fiber. The CCD detector array (Hamamtsu, Inc.) in the spectrometer has a fast electronic shutter, enabling fast gating of the spectral measurement, which minimizes the effects of background light. Diffusely reflected light from the tissue is transmitted by the collection fiber and coupled directly to the entrance slit of the spectrometer. The fiberoptic probe used in these studies (Fig. 6) consisted of a 150-μm-core diameter illumination fiber and an adjacent 100-μm-diameter collection fiber, with center-to center separation of ~150 microns. For the *ex-vivo* measurements reported here, the diameter of the outer tubing of the fiberoptic handpiece was ~1 mm. (In future studies of *in-vivo*, transcutaneous measurements, smaller fibers will be incorporated into a 25-gauge fine needle, to minimize tissue trauma).

Prior to recording spectra from the tissue, a calibration spectrum from a spectrally-flat diffuse reflection standard (Spectralon^TM^: Labsphere, Inc.) is taken, *I*(*λ*)_*ref*_. The diffuse reflectance of this reference material is spectrally flat when measured at a short distance from the surface (>98% over the full spectral range of the ESS measurement). The reference spectrum is used to account for any spectral variations in the light source, spectrometer, fiber transmission and fiber coupling. All spectra in this study were recorded as ratios of the tissue spectrum to the reference spectrum, providing spectra that are independent of the spectral response of the system. As such, the optical system, including the probe, was calibrated at the time of the measurement, and the tip of the probe was then placed in contact with the tissue site to be investigated. Spectral measurements were triggered by the use of either a foot pedal or the keyboard on the integrated computer. Even though the fast optical gating of the detector results in minimal effects of background light, the instrumentation compensates for any background light (and electronic detector offset) by taking an initial measurement without firing the xenon lamp [*I*(*λ*)_*tissue background*_ or *I*(*λ*)_*ref background*_] followed immediately by a subsequent reference or tissue measurement with the pulsed lamp triggered. The calculated ESS spectra are the result of the subtraction of the background spectrum from the tissue or reference spectrum. Thus, calibrated spectra were calculated according to:

$$I(\lambda )=\frac{I{(\lambda )}_{tissue}-I{(\lambda )}_{tissuebackground}}{I{(\lambda )}_{ref}-I{(\lambda )}_{refbackground}}$$

### Calculation of spectral biomarker

The spectra were all normalized to a value 1 at 650 nm, and the area of the spectrum from 350–450 nm was determined simply by adding the values of all the pixels in that range. Given the normalization, spectral area values are relative and can be scaled arbitrarily for presentation.

### Animal models for chronic kidney disease

All the experiments were conducted after approval of the Institutional Animal Care and Use Committee of Boston University School of Medicine. (IACUC approved protocol # AN-15449) All methods were performed in accordance with the relevant guidelines and regulations established by that governing committee.

#### UUO model

Briefly, a group of 12-week-old C57BL/6 mice underwent laparotomy, and the left ureter was ligated close to the bladder. Four animals were sacrificed once a week for 4 weeks (plus time t = 0), and both the obstructed and the contralateral kidneys were isolated. The kidneys were sliced through the hilum along their vertical axes. The ESS spectra were obtained using the fiberoptic probe on several points on the surface, cortex and the medulla of the kidneys. ESS spectra obtained from time t = 0 served as controls. After ESS measurements, the sliced kidneys were paraffin-embedded and stained with Masson Trichrome, which stains extracellular matrix, especially collagen-I.

#### The adenine-diet-induced (AD) mouse model

A group of both C57BL/6 and 129/SvEv strains of male and female mice (n = 10) was fed with a customized diet containing normal chow (Teklad Global 18% Protein Rodent Diet) mixed with 0.25% adenine (Research Diets, NJ) for one week followed by 0.20% diet for 3 weeks^59^. A separate group (n = 10) on normal chow served as controls. After euthanasia, the kidneys and blood were processed and ESS measurements taken, as described above.

### Histological evaluation

Hematoxylin & Eosin and Masson Trichrome stains were performed on the slides in the Histopathology core at Boston University Medical Center. The slides were evaluated by a nephropathologist for the following features.

Tubulointerstitial: tubular atrophy, dilatation, casts, interstitial inflammation, and fibrosis as parameters of interstitial damage were determined using a semi-quantitative IFTA scoring method on PAS stained sections. IFTA Grade 0 indicates no changes; grade 1, lesions involving less than 25%; grade 2, lesions affecting 25–50%; and grade 3, affecting 50–75%; grade 4 lesions involving more than 75% of the field^60–62^.

## Data Availability

The spectral files reported and analyzed in this report are available to academic researchers upon request to the corresponding author.
